# Combination of an ACLY inhibitor with a GLP-1R agonist exerts additive benefits on nonalcoholic steatohepatitis and hepatic fibrosis in mice

**DOI:** 10.1016/j.xcrm.2023.101193

**Published:** 2023-09-19

**Authors:** Eric M. Desjardins, Jianhan Wu, Declan C.T. Lavoie, Elham Ahmadi, Logan K. Townsend, Marisa R. Morrow, Dongdong Wang, Evangelia E. Tsakiridis, Battsetseg Batchuluun, Russta Fayyazi, Jacek M. Kwiecien, Theodoros Tsakiridis, James S.V. Lally, Guillaume Paré, Stephen L. Pinkosky, Gregory R. Steinberg

**Affiliations:** 1 Centre for Metabolism Obesity and Diabetes Research, McMaster University, Hamilton ON L8S 4L8, Canada; 2 Division of Endocrinology and Metabolism, Department of Medicine, McMaster University, Hamilton, ON L8S 4L8, Canada; 3 Department of Pathology, McMaster University, Hamilton, ON L8S 4L8, Canada; 4 Department of Oncology, McMaster University, Hamilton, ON L8S 4L8, Canada; 5 Population Health Research Institute, McMaster University, Hamilton, ON L8L 2X2, Canada; 6 Thrombosis and Atherosclerosis Research Institute, McMaster University, Hamilton, ON L8L 2X2, Canada; 7 Esperion Therapeutics, Inc., Ann Arbor, MI, USA; 8 Department of Biochemistry and Biomedical Sciences, McMaster University, Hamilton, ON L8L 2X2, Canada

**Keywords:** NAFLD, NASH, hepatic fibrosis, ACLY, bempedoic acid, GLP-1R agonist, liraglutide, combination treatment, mouse model, semaglutide, MASH, lipogenesis, fatty acid metabolism

## Abstract

Increased liver *de novo* lipogenesis (DNL) is a hallmark of nonalcoholic steatohepatitis (NASH). A key enzyme controlling DNL upregulated in NASH is ATP citrate lyase (ACLY). In mice, inhibition of ACLY reduces liver steatosis, ballooning, and fibrosis and inhibits activation of hepatic stellate cells. Glucagon-like peptide-1 receptor (GLP-1R) agonists lower body mass, insulin resistance, and steatosis without improving fibrosis. Here, we find that combining an inhibitor of liver ACLY, bempedoic acid, and the GLP-1R agonist liraglutide reduces liver steatosis, hepatocellular ballooning, and hepatic fibrosis in a mouse model of NASH. Liver RNA analyses revealed additive downregulation of pathways that are predictive of NASH resolution, reductions in the expression of prognostically significant genes compared with clinical NASH samples, and a predicted gene signature profile that supports fibrosis resolution. These findings support further investigation of this combinatorial therapy to treat obesity, insulin resistance, hypercholesterolemia, steatohepatitis, and fibrosis in people with NASH.

## Highlights

• Inhibiting liver ATP citrate lyase (ACLY) lowers cholesterol, steatosis, and fibrosis
• GLP-1 receptor (GLP-1R) agonists lower body mass, glucose, and liver steatosis
• In mice, combining a GLP-1R agonist and an ACLY inhibitor reduces NASH
• Transcriptional signature of combo therapy is associated with fibrosis resolution

## Graphical abstract

**Figure:**
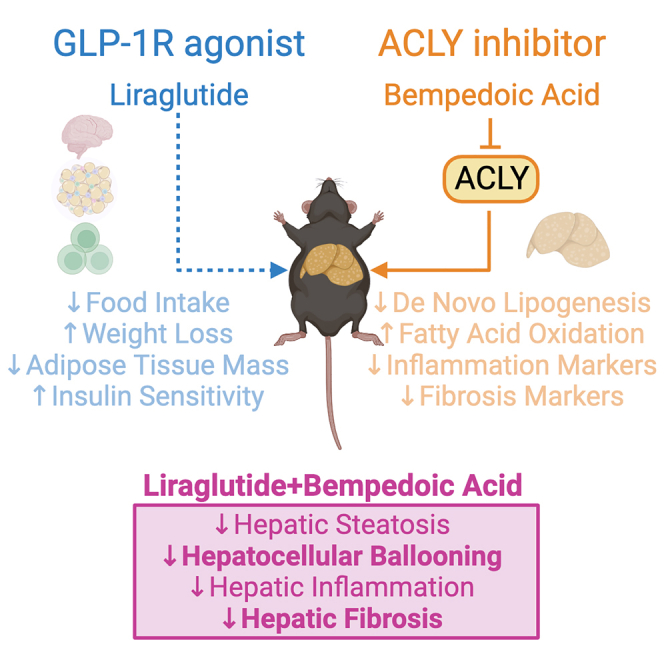

## Teaser

Desjardins et al. find that combination therapy in mice with the ACLY inhibitor bempedoic acid and the GLP1-R agonist liraglutide leads to greater reductions in NASH and fibrosis than monotherapies and is associated with transcriptional signatures consistent with the resolution of liver fibrosis in clinical populations.

## Introduction

The rising prevalence of both adult and childhood nonalcoholic fatty liver disease (NAFLD) poses immense burden on patients’ health-related quality of life and healthcare costs.^1^ The advanced form of NAFLD—nonalcoholic steatohepatitis (NASH)—is typically characterized by liver steatosis, hepatocellular ballooning, and lobular inflammation with or without perisinusoidal fibrosis and can manifest itself to cirrhosis, liver failure, cancer, and death.^2^^,^^3^ Currently, there are no approved therapies for the treatment of NASH. In human clinical trials, monotherapies tested in phase 3 trials have not reached primary endpoint goals for both reductions in the NAFLD activity score and fibrosis or have been called into question as to whether benefit outweighs risk.^4^ Bearing in mind that almost half of the people with NAFLD also live with an associated metabolic comorbidity (69% hyperlipidemia, 51% obesity, 39% hypertension, 22% type 2 diabetes, and 42% metabolic syndrome),^5^ it is reasonable to suspect that combinatorial therapies that target different aspects of NASH pathophysiology and associated comorbidities could lead to improved efficacy in treating NASH, secondary measures, and tolerability, which could sway the risk-benefit ratio.^6^^,^^7^

Glucagon-like peptide-1 receptor (GLP-1R) agonists have been studied in clinical trials for NASH.^8^^,^^9^^,^^10^^,^^11^ GLP-1 is an incretin hormone that is secreted in the postprandial phase by intestinal L cells to control blood glucose, satiety, and gastrointestinal motility.^7^ Liraglutide is a long-acting GLP-1R agonist that is approved for the treatment of both type 2 diabetes and obesity.^12^ The combination of liraglutide and exercise also leads to greater maintenance of weight loss,^13^ abdominal fat, and markers of inflammation^14^ than either treatment alone. In a multicenter, randomized, placebo-controlled trial, liraglutide treatment resulted in greater NASH resolution (39%) versus placebo (9%).^10^ Semaglutide, a long-acting GLP-1R agonist similar to liraglutide but with more pronounced weight loss effects, showed an even higher percentage of NASH resolution (59%, 0.4 mg dose) versus placebo (17%) in a 72 week double-blinded phase 2 trial with biopsy-confirmed NASH and liver fibrosis of stages F1–F3.^8^ However, in a recent trial, semaglutide did not improve fibrosis or NASH resolution versus placebo in patients with NASH-related cirrhosis.^11^ These data indicate that while GLP-1R agonists can effectively reduce body mass—in turn reducing liver steatosis—and may have some anti-inflammatory effects by acting on a small population of liver-localized γδ T cells, they do not appear to exert anti-fibrotic effects.^15^^,^^16^ This is likely because the GLP-1R is not expressed in hepatocytes, Kupffer cells, or hepatic stellate cells and therefore does not directly act on the key cells driving fibrosis.^17^

Increases in liver *de novo* lipogenesis (DNL) are a hallmark of patients with NAFLD.^18^^,^^19^^,^^20^ A critical enzyme controlling flux through the DNL pathway is ATP citrate lyase (ACLY), which synthesizes acetyl-CoA and oxaloacetate from citrate (reviewed in Nissen et al.^21^). Bempedoic acid is a small-molecule ACLY inhibitor that reduces risk of major adverse cardiovascular events in statin-intolerant patients and has been approved by the US Food and Drug Administration (FDA) and the European Commission (EC) to reduce low-density lipoprotein cholesterol (LDL-C) in adults with heterozygous familial hypercholesterolemia or established atherosclerotic cardiovascular disease.^21^^,^^22^^,^^23^^,^^24^^,^^25^^,^^26^ Bempedoic acid is a prodrug that is converted to its active moiety, bempedoyl-CoA, in the liver by the very-long-chain acyl-CoA synthetase 1 (ASCVL1).^27^ This liver-targeted conversion is important to minimize muscle-related side effects, which is associated with the use of statins.^27^ In a mouse model of NASH that has very similar metabolic (i.e., obesity, insulin resistance), histological, and transcriptional characteristics to people with advanced NASH that is induced by housing male mice at thermoneutrality (∼29°C) and feeding a high-fat diet supplemented with fructose and physiological concentrations of cholesterol,^28^ we have found that bempedoic acid reduced liver steatosis, hepatocellular ballooning, lobular inflammation, and also fibrosis.^28^ Additional experiments in hepatic stellate cells from mice and humans demonstrated that bempedoic acid suppressed lipogenesis and blocked transforming growth factor β (TGF-β)-induced proliferation and activation.^28^ Importantly, bempedoic acid also exerted pronounced anti-fibrotic effects independently of reductions in steatosis in the STAM mouse model of NASH, which does not have obesity or insulin resistance.^28^ Taken together, these data suggest that the beneficial effects of bempedoic acid on NASH and fibrosis are likely to be completely distinct from the primary pathways targeted by liraglutide, suggesting that there may be additive effects combining the two therapeutics.

Considering the distinct mechanisms by which liraglutide and bempedoic acid reduce NAFLD, the purpose of this study was to evaluate whether the addition of bempedoic acid to liraglutide would elicit added benefits to treating NASH and hepatic fibrosis in a physiologically relevant mouse model that replicates many of the metabolic, histological, and transcriptional characteristics of patients with advanced NASH.

## Results

### Combination of liraglutide and bempedoic acid reduces body weight, adiposity, glucose intolerance, insulin resistance, and serum cholesterol

As we have previously described, housing C57BL/6J mice at thermoneutrality and feeding a diet high in fat and fructose leads to metabolic, pathological, and transcriptional characteristics similar to human NASH.^28^ Using this diet and housing paradigm, after 16 weeks, mice were assigned to five interventional arms by matching body weight and adiposity so that there were no differences at the start of the treatment period.

Consistent with our recent study,^28^ bempedoic acid (BemA) was mixed with the diet at a concentration of 10 mg/kg, and its effects on the mice were compared with mice that received the same diet minus BemA (control). BemA did not alter body weight, adiposity, glucose tolerance, insulin sensitivity, pyruvate tolerance (a measure of hepatic gluconeogenesis), fasting serum insulin, or triglyceride levels but did reduce serum cholesterol (Figures S1A–S1H). Despite similar adiposity and glucose homeostasis, BemA reduced liver fat percentage, pathological scoring of liver steatosis, hepatocellular ballooning, and the NAFLD activity composite score (Figures S1I–S1O). Importantly, BemA also reduced the percentage of fibrosis area assessed using picrosirius red (PSR) and the presence of moderate, zone 3 perisinusoidal fibrosis (2 of 9) assessed by a pathologist compared with control mice (4 of 9) (Figures S1P and S1Q). These data indicate that, consistent with our previous study^28^ using thermoneutral housing but a shorter duration of dietary intervention before initiating treatment (10 versus 16 weeks in the current study), BemA reduces liver steatosis, ballooning, and fibrosis independently of changes in body mass or adiposity.

**Figure 1:**
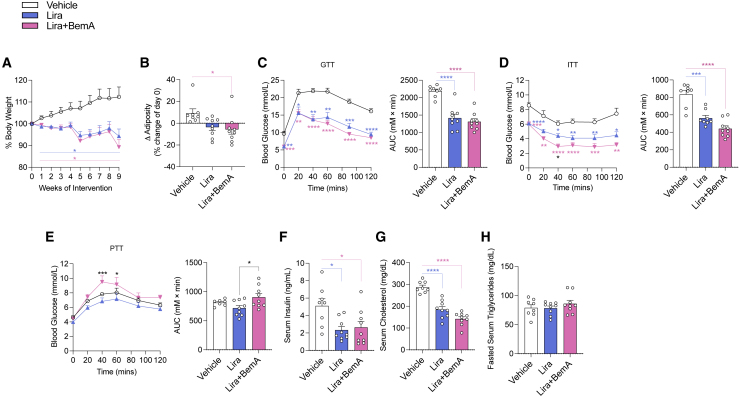
Lira and BemA lower body mass, adiposity, insulin sensitivity, and serum cholesterol without increasing serum triglycerides (A and B) Percentage change in body weight (A) and change in adiposity (post-pre) (B) throughout intervention. (C–E) Intraperitoneal glucose tolerance test (GTT) (1.25 g/kg) (C) at 4 week intervention, intraperitoneal (i.p.) insulin tolerance test (ITT) (1.3 U/kg) (D) at 4 week intervention, and i.p. pyruvate tolerance test (PTT) (1.5 g/kg) (E) at 5 week intervention with time plots and area under the curve (AUC). (F) Fasted serum insulin collected via tail nick near end of intervention (9 weeks). (G and H) Fed serum cholesterol (G) from blood collected by cardiac puncture at sacrifice and fasted serum triglycerides (H). Data are means ± SEM. Colored bars signify comparisons between groups and vehicle. Significance was accepted at p <0.05 and determined via one-way ANOVA or repeated-measures two-way ANOVA with Tukey post hoc, where appropriate. White circles are individual mice per group (n = 8–9 mice/group). ∗p < 0.05, ∗∗p < 0.01, ∗∗∗p < 0.001, ∗∗∗∗p < 0.0001. Vehicle (saline treatments subcutaneously every 2 days), Lira (70 μg/kg Lira subcutaneously every 2 days before lights out), and Lira+BemA (BemA 10 mg/kg in diet and 70 μg/kg Lira subcutaneously every 2 days before lights out).

Modest reductions in body mass can completely resolve NASH in mouse models.^29^ Treatment of mice with GLP-1R agonists such as liraglutide (Lira) dose dependently suppress appetite and body mass and can, at higher doses, reduce body mass by greater than 25% over just 14 days of treatment after 8 weeks of a high-fat diet.^30^ Human clinical trials with Lira in people with NASH elicit 5%–10% weight loss.^10^ Therefore, to enhance the potential translatability of Lira treatment in mice to humans with NASH, we utilized a submaximal dose of Lira with the aim to elicit similar reductions in body mass/adiposity to that observed in participants within clinical trials. Consistent with this aim, treatment of mice with Lira compared with mice injected at the same frequency with vehicle control reduced body mass by 6% at 9 weeks of treatment, and this effect on body mass was not altered by the addition of BemA (Lira+BemA) (Figures 1A and 1B). Compared with vehicle control mice, Lira and Lira+BemA improved glucose tolerance (Figure 1C) and insulin sensitivity (Figure 1D) but did not alter pyruvate tolerance (Figure 1E). Lira lowered fasting serum insulin (Figure 1F) and cholesterol levels (Figure 1G), and these effects were also observed in mice treated with Lira+BemA. Serum triglycerides (Figure 1H) were unchanged in either Lira or Lira+BemA combination groups.

### Combination of Lira and BemA results in additive benefits on liver steatosis, ballooning, and fibrosis

**Figure 2:**
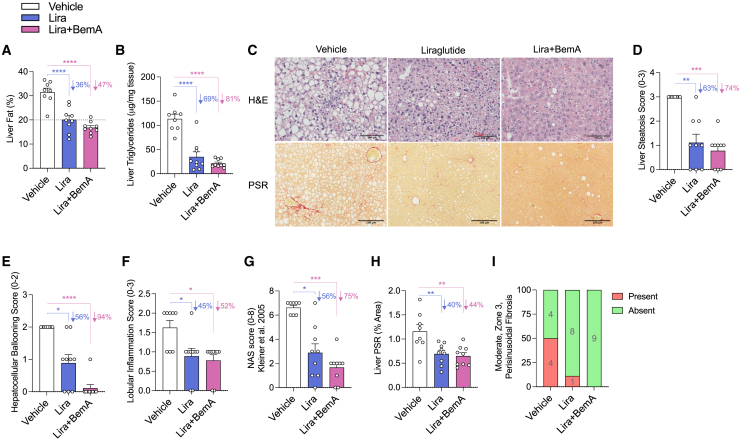
Lira and BemA reduce liver steatosis, ballooning, inflammation, and fibrosis (A) Liver fat percentage as measured by time-domain nuclear magnetic resonance (NMR). (B) Liver triglycerides. (C–H) Representative micrographs of H&E- (top) and picrosirius red- (PSR; bottom) stained sections (10×) (C) along with histograms of histological grades of liver steatosis (D), hepatocellular ballooning (E), lobular inflammation (F), and composite NAFLD activity score (NAS) (G). (H and I) Percentage of positive PSR area (H) and parts of whole indicating presence of moderate, zone 3 perisinusoidal fibrosis (I). Data are means ± SEM. Colored bars signify comparisons between groups and vehicle with percentages listed next to them. Significance was accepted at p <0.05 and determined via one-way ANOVA with Tukey post hoc, or, for histological score analysis, a Kruskal-Wallis test was used with Dunn’s post hoc test to correct for multiple comparisons, where appropriate. White circles are individual mice per group (n = 8–9 mice/group). ∗p < 0.05, ∗∗p < 0.01, ∗∗∗p < 0.001, ∗∗∗∗p < 0.0001. Vehicle (saline treatments subcutaneously every 2 days), Lira (70 μg/kg Lira subcutaneously every 2 days before lights out), and Lira+BemA (BemA 10 mg/kg in diet and 70 μg/kg Lira subcutaneously every 2 days before lights out).

In comparison to the vehicle group, Lira and Lira+BemA reduced the percentage of liver fat by 36% and 47%, respectively (Figure 2A), and triglycerides by 69% and 81%, respectively (Figure 2B). Consistent with these observations, steatosis scores from H&E sections were reduced with Lira (63%) and Lira+BemA treatments (74%) (Figures 2C and 2D). Hepatocellular ballooning scores were reduced with Lira by 56% and with Lira+BemA by a remarkable 94% (Figures 2C and 2E). Lira and Lira+BemA reduced lobular inflammation scores to a similar degree (∼50%) (Figures 2C and 2F). In sum, the NAFLD activity score (NAS) was reduced by 56% by Lira and by 75% by Lira+BemA (NAS; Figure 2G). Importantly, Lira and Lira+BemA treatment groups reduced fibrosis area assessed using PSR (40% and 44%, respectively) (Figures 2C and 2H) and had fewer (Lira: 1 of 9) or no (Lira+BemA: 0 of 9) moderate zone 3 perisinusoidal fibrosis compared with vehicle-treated mice (4 of 8) (Figures 2C and 2I).

Serum biomarkers of liver inflammation/damage in clinical populations including alanine transaminase (ALT), aspartate aminotransferase (AST), serum amyloid A (SAA), the C-X-C motif chemokine ligand 10 (CXCL10), C-reactive protein (CRP), and secreted phospholipase A2 (sPLA2) were measured. BemA reduced ALT, AST, SAA, CXCL10, CRP, and sPLA2 compared with control, while Lira significantly reduced SAA and sPLA2 versus vehicle. Combination of Lira+BemA reduced SAA similarly to Lira alone, while it resulted in a significant reduction in CRP in comparison to vehicle. Finally, Lira+BemA significantly reduced sPLA2 in comparison to both vehicle and Lira groups (Table 1).

**Table 1:** Serum biomarkers in mice treated with vehicle, Lira, BemA, or Lira+BemA

	Group (mean ± SEM)								
	Vehicle		Lira		Lira+BemA		Control	BemA	
Measure	n = 8	p value	n = 9	p value	n = 9	p value	n = 9	n = 9	p value
ALT (U/mL)	230.4 ± 31.1	0.4017	337.5 ± 90.1	0.5967	356.2 ± 85.5	0.4936	280 ± 46.5	33.5 ± 19.4	0.0002
AST (U/mL)	63.3 ± 7.3	0.0598	128.4 ± 30.6	0.1891	144.1 ± 28.5	0.0846	92.7 ± 11.9	28.4 ± 4.3	0.0001
SAA (ng/mL)	2743 ± 60	0.8786	1,092 ± 248	0.0004	1,154 ± 331	0.0005	2,730 ± 49	1,064 ± 176	<0.0001
CXCL10 (pg/mL)	11.6 ± 1.4	0.6811	10 ± 0.6	0.4470	8.4 ± 0.9	0.0689	12.7 ± 1.9	7.3 ± 0.5	<0.0001
CRP (pg/mL)	29,169 ± 5,539	0.7668	18,033 ± 2,133	0.0634	12,227 ± 983	0.0038	31,873 ± 6,845	15,664 ± 3,074	0.0463
sPLA2 (nmol/min/mL)	26.9 ± 1.1	0.6052	20.5 ± 0.5	0.0003	16.3 ± 1.1	<0.0001^a^	28 ± 1.7	19.3 ± 0.8	0.0003

p values reported are based on comparisons used throughout the remainder of manuscript—unpaired t test between control and BemA and one-way ANOVA with Tukey post hoc between vehicle, Lira, and Lira+BemA. Significance was accepted at p <0.05. n = 8–9 mice/group. ALT, alanine transaminase; AST, aspartate aminotransferase; SAA, serum amyloid A; CXCL10, C-X-C motif chemokine ligand 10, also known as interferon gamma-induced protein 10 (IP-10); CRP, C-reactive protein; sPLA2, secretory phospholipase A2.

a Significant difference p <0.05 between Lira and Lira+BemA.

Collectively, Lira+BemA led to greater percent reductions and lower p values for steatosis, ballooning, NAS, PSR, and sPLA2 compared with Lira monotherapy. Notably, Lira+BemA almost completely attenuated hepatocyte ballooning, and this was the primary contributor to the greater percentage reduction in NAS with combination treatment. These data suggest BemA may have additive effects with Lira toward improving liver pathology.

### Targeted gene expression profiling identifies additive downregulation of fibrosis-related molecular pathways that are predictive of NASH resolution

**Figure 3:**
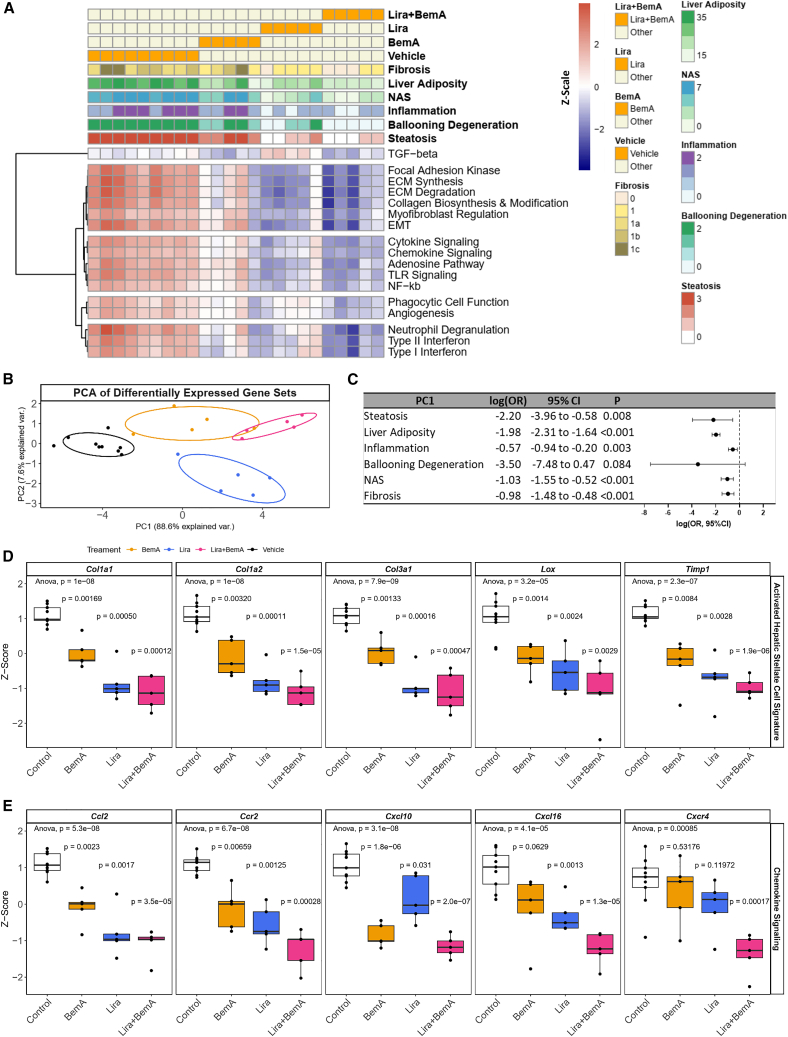
Gene set analysis reveals additive downregulation of fibrosis-related pathways by combination treatment that are associated with NASH resolution (A) Signature scores of transcriptional pathways most affected by combination treatment. Each heatmap column represents an individual sample, along with rows annotated according to treatment cohort, histology, and gene set scores. (B) PCA of control, monotherapy, and combination treatments based on gene set scores. (C) Odds ratio and 95% confidence interval associated with hepatic steatosis, ballooning degeneration, inflammation, fibrosis, NAS, and liver adiposity measurements based on PC1 of gene set scores. (D and E) Gene expression of hepatic stellate cell markers (D) and chemokines (E) associated with NASH progression. Black circles are individual mice per group (n = 5–9 mice/group). Black circles represent individual mice per group (n = 5–9 mice/group). Boxplots show median and interquartile range, trailing lines represent 95% confidence interval. Difference between groups were assessed by one-way ANOVA followed by Dunnett’s post hoc test using the control group as the reference level. Significance was accepted at p < 0.0033 to correct for Bonferroni multiple hypothesis testing.

To determine the transcriptional differences between our treatment cohorts, we examined the expression of 760 genes implicated in 49 fibrosis-related pathways using the nCounter Fibrosis v.2 Panel. Differential expression analysis comparing Lira, BemA, and combination treatment to vehicle controls yielded 249, 132, and 263 differentially expressed genes, respectively (Figures S2A; Table S1). Combination treatment resulted in the greatest number of downregulated genes, significantly reducing the expression of 172 genes compared with 97 and 86 by Lira and BemA alone, respectively. Of these, 56 genes were uniquely altered by combination treatment, 113 genes overlapped between all treatment cohorts, and 3 genes were upregulated by Lira but downregulated in the combination cohort (Figures S2B; Table S2). Conversely, 8 genes were uniquely upregulated by combination treatment (Figure S2C). Over-representation analysis of the uniquely downregulated and overlapping genes, which we defined as additive if the effect size was largest in the combination treatment cohort, identified seven disease processes of interest related to inflammation, fibrosis, and wound healing (Figures S2D; Table S3). Next, we utilized a more comprehensive approach to identify gene sets altered by combination treatment by using all genes in the nCounter panel (Figure S2E).^31^ Combination treatment led to reductions across 17 pathways with hierarchical clustering identifying reductions in overarching disease processes related to fibrosis (e.g., collagen biosynthesis and modification, myofibroblast regulation), inflammation (e.g., chemokine signaling, cytokine signaling), and wound healing (e.g., phagocytic cell function, angiogenesis), which was consistent with pathway annotation analysis (Figure 3A).

To assess gene set association with phenotype observations, we regressed disease outcome measurements on the first principal component (PC1) of the 17 gene sets (Figure 3B) most affected by combination treatment relative to monotherapy. This demonstrated a significant predictive relationship between PC1 increment and hepatic steatosis, inflammation, fibrosis, adiposity, and NAS resolution (Figure 3C).

Hepatic stellate cells are critical for driving liver fibrosis, and therefore we explored the expression of key markers implicated in NASH progression.^32^ Consistently, markers of activated stellate cells (*Col1a1*, *Col1a2*, *Col3a1*, *Lox*, *Timp1*) were significantly reduced in the Lira+BemA treatment groups to a greater extent than monotherapies of Lira or BemA (Figure 3D). Interestingly, BemA appeared to counteract Lira-induced upregulation of TGF-β effectors, including *Smad3*, a transcription factor critical for upregulating fibrotic pathways in NASH (Figure S2F). However, there was no change in the phosphorylation of SMAD 2/3 compared with vehicle controls (Figure S2G). Moreover, combination therapy generally reduced the expression of several chemokines implicated in NASH progression greater than Lira or BemA treatment alone (Figure 3E). Collectively, these data indicate that combination therapy with Lira+BemA induces an anti-fibrotic and anti-inflammatory gene expression profile that is predictive of reduced inflammation, ballooning, and fibrosis.

### Combination treatment induces a prognostically favorable gene expression profile that most closely resembles those from healthy human liver biopsies

**Figure 4:**
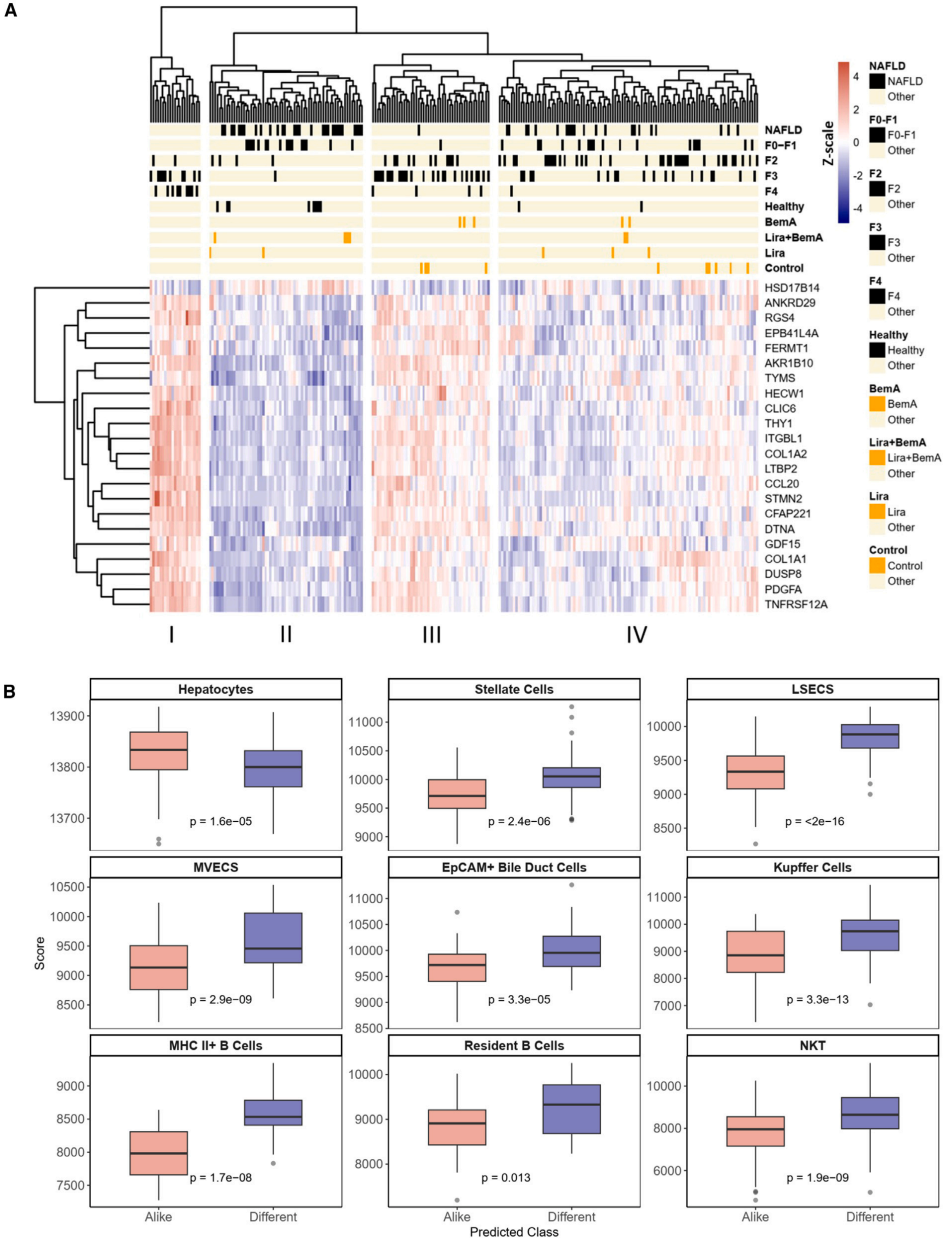
Combination treatment promotes gene expression associated with NASH resolution (A) Hierarchical clustering of healthy human patients, patients with NAFLD/NASH, and experimental cohorts based on the standardized expression of prognostically significant orthologous genes involved in NASH progression. (B) Classification of human-derived samples based on similarity to combination treatment gene signature identifies differential enrichment of parenchymal and nonparenchymal cell types between predicted classes. Boxplots show median and interquartile range, trailing lines represent 95% confidence interval. Outliers are represented by black circles. Difference between groups were assessed by Students’ t test and significance was accepted at p < 0.0056 to correct for Bonferroni multiple hypothesis testing.

In humans, a 25-gene signature has been established to be predictive of NASH severity.^33^ Therefore, to contextualize the clinical significance of our experimental therapies, we performed an integrative analysis combining the expression data of 22 orthologous genes derived from our treatment cohorts with the expression data derived from 216 patients with NAFLD/NASH. Combination treatment significantly downregulated the expression of 13 genes in this prognostic signature (Figure S3A). Hierarchical clustering using Pearson correlation reveals four clusters with differential compositions of healthy individuals, patients with prefibrotic (NAFLD, F0–F1) or fibrotic (F2–F4) disease and our experimental cohorts (Figure 4A). Cluster II exhibits the most clinically benign phenotype, with 80% of healthy individuals in the patient-derived dataset represented in this cluster compared with 7.55%, 1.85%, and 0% of patients with F2, F3, and F4 stages of disease (Figure S3B). We find that 4 out of 6 of our combination treatment samples colocalized in this cluster, while monotherapy treatment samples are mostly grouped in clusters I and II, which exhibit more advanced disease. Using PC analysis (PCA), we show the progressive resolution of NASH in human patients on PC1 (Figure S3C). Mapping our control, monotherapy, and combination treatment cohorts with human NASH disease stages based on PC1 and PC2 further supports the increased transcriptional similarity between healthy individuals and combination treatment samples beyond what can be achieved using Lira and BemA alone.

Classification of patients into disease subtypes based on their expression of predefined gene signatures can inform risk stratification and elucidate molecular characteristics between disease subtypes. Recent studies have similarly utilized treatment-specific gene signatures to classify patients exhibiting similar or dissimilar gene expression profiles into treatment-responsive or nonresponsive subtypes.^34^^,^^35^ This analysis may provide preliminary evidence informing clinical translation and elucidate molecular differences driving therapeutic response in patients. As such, we derived a combination-treatment-specific gene signature by filtering for genes that were differentially regulated by combination treatment when compared with both control and monotherapy (Figures S4A and S4B).

To determine the prognostic significance of this gene signature, we first compared its predictive performance using multivariate logistic regression to the reference gene signature derived by Govaere et al. in the human NAFLD/NASH cohort. For the prediction of fibrosis stages >2, the treatment signature achieved an area under receiver operating curve (AUROC) score of 0.899, which approximates the score of 0.922 achieved by the reference signature (Figure S4C). Further variable selection using elastic net regularization did not improve prediction accuracy (AUROC = 0.893). Among the signature genes, *AXL*, *SLC2A2*, *CYBB*, *DOCK2*, *C3AR1*, *CYFIP1*, and *LEPR* were significantly associated with advanced fibrosis in the multivariate model. Subsequently, we utilized nearest template prediction to classify patients based on the similarity of their gene expression profile to our treatment signature.^36^ Patients classified as similar predominantly exhibited prefibrotic stages of disease (NAFLD and F0–F1), whereas those classified as dissimilar were enriched for fibrotic stages (F2–F4) (Figure S4D). Single-sample gene set enrichment analysis (ssGSEA) of hallmark gene sets and liver cell types reveal a favorable transcriptional profile for NASH resolution. Patients similar to our treatment gene signature exhibit significant downregulation of fibrosis-, inflammation-, and cellular-damage-related gene sets, while pathways related to fatty acid metabolism, oxidative phosphorylation, and DNA repair are significantly upregulated (Figure S4E). Correspondingly, liver cell-type analysis based on markers derived from the human liver cell atlas^37^ reveals significant downregulation of all nonparenchymal liver cell types including hepatic stellate cells and liver sinusoidal endothelial cells and, conversely, upregulation of hepatocytes (Figures 4B and S4F). Overall, the gene expression pattern identified in patients who exhibit a similar gene signature profile to combination treatment supports fibrosis, steatosis, and inflammation resolution among patients with NAFLD/NASH.

## Discussion

In this study, we combined two approved treatments that have distinct molecular targets, have excellent safety profiles, and treat separate comorbidities associated with NAFLD. Lira caused reductions in adiposity, glucose intolerance, and insulin resistance, while BemA treatment resulted in reductions in total cholesterol, consistent with both drugs’ primary approved indication: type 2 diabetes/obesity and cardiovascular disease, respectively. With respect to liver pathology, both BemA and Lira monotherapies lowered steatosis, ballooning, and fibrosis to a similar degree, and we found that when combined, there were greater percentages of reductions in hepatocellular ballooning and fibrosis.

DNL is an important cause of liver steatosis in people with NAFLD.^38^^,^^39^ ACLY sits upstream of acetyl-CoA carboxylase (ACC) in the DNL pathway, generating acetyl-CoA and oxaloacetate, and is inhibited with BemA.^28^ We found that, consistent with previous observations,^28^^,^^40^ both Lira and BemA reduced liver fat percentage, triglycerides, steatosis scores, and, in contrast to ACC inhibitors,^4^ did not result in elevated serum triglycerides. The combination of both Lira and BemA led to a greater percentage of reductions in steatosis scores and liver triglycerides, an effect that was likely due to differential mechanisms of action. Specifically, Lira-induced weight loss is known to improve adipose tissue insulin sensitivity,^41^ which is expected to reduce lipolysis and hepatocyte acetyl-CoA levels,^42^ while BemA directly inhibits acetyl-CoA formation from citrate.^43^ Collectively, this bimodal mechanism of action would be expected to reduce malonyl-CoA, leading to increases in fatty acid oxidation, reductions in DNL, and ultimately steatosis, as observed in the current study.

Cellular ballooning is one of the principle histological findings used to identify the presence of steatohepatitis in NASH.^44^ The most striking histological observations with Lira+Bema combination therapy, compared with either monotherapy alone, was the dramatic reduction in hepatocyte ballooning. This reduction in ballooning was the primary driver for the reduction in NAS. The mechanisms driving hepatocyte ballooning have been attributed to increases in lipid oxidative stress, endoplasmic reticulum and cytoskeletal dysfunction, and impaired autophagy.^44^ As hepatocytic ballooning has been associated with greater severity of liver disease and a higher risk of developing liver-related complications, cardiovascular disease, and chronic kidney disease in NASH,^44^ future studies investigating the mechanisms of how Lira and Bema reduce ballooning are warranted.

The prominent feature differentiating steatohepatitis from liver steatosis is the development of inflammation. Our transcriptome analysis indicates that the combination of BemA and Lira reduced inflammation as characterized by cytokine and chemokine signaling, the adenosine pathway, Toll-like receptor (TLR) and nuclear factor κB (NF-κB) signaling, phagocytic cell function, neutrophil degranulation, and interferon signaling. Furthermore, the reductions in inflammatory markers within the liver were more broadly apparent in the measurements of systemic markers of inflammation in serum, as shown by large reductions in SAA. The additive effects of BemA and Lira on reducing inflammatory transcripts are consistent with previous studies in which exercise training in combination with Lira also led to greater reductions in abdominal obesity and inflammation then either therapy alone.^14^

We found that combining BemA with Lira reduced fibrosis. These pathological findings were supported by transcriptomic data indicating that combination therapy reduced extracellular matrix synthesis, epithelial-to-mesenchymal transition, myofibroblast regulation, focal adhesion kinase, and collagen biosynthesis and modification. This is important because neither Lira nor semaglutide have shown efficacy at reducing fibrosis.^8^^,^^10^^,^^11^ Mechanistically, our transcriptomic analyses revealed that a potentially unique effect of BemA may be due to its effects on countering Lira-induced increases in the TGF-β-activated transcription factor *Smad3*, which is a critical driver of fibrosis.^45^ As the GLP-1R is not expressed on hepatic stellate cells,^17^ and BemA treatment attenuates TGF-β-mediated activation of both murine and human hepatic stellate cells *in vitro*,^22^ this direct effect on hepatic stellate cells may be important for reducing fibrosis. However, further studies evaluating the effects of genetically inhibiting ACLY in hepatic stellate cells will be required to fully interrogate this hypothesis.

Given the complexity of NASH pathophysiology, targeting multiple distinct pathways may be required to confer clinical improvements not only in liver steatosis, inflammation, and fibrosis but also in associated comorbidities that are more likely to cause death to patients living with the disease.^5^ This is supported by clinical studies illustrating that exercise and Lira lead to greater weight loss, visceral adiposity, and inflammation compared with either therapy alone.^13^^,^^14^ There are several other rationales for the use of combination therapies to treat NASH, as combining two or more drugs could increase response rates, maximize response to treatment, reduce side effects, and address loss of effects over time.^6^ And although combinatorial therapies may increase the difficulty of designing trials and present more challenges in patient recruitment/retainment, these efforts may improve the benefit:risk ratio compared with monotherapies.

In summary, the current study demonstrates that combining BemA with Lira leads to greater percentage of reductions in liver pathology, most notably ballooning and fibrosis, compared with monotherapy in a mouse model of metabolic-associated NASH. These findings support further investigation and potential development of this combinatorial therapy to treat obesity, insulin resistance, hypercholesterolemia, steatohepatitis, and fibrosis in people with NASH.

### Limitations of the study

Our data demonstrate a benefit of combining the ACLY inhibitor BemA and the GLP-1R agonist Lira; however, this study has several limitations. Our experimental design and analyses were not able to detect statistically significant differences in pathological scoring between Lira and Lira+BemA, although the percentage of reduction in most parameters was consistently greater with the combination treatment regime. This finding is similar to other preclinical studies that have also tested combination therapies with other GLP-1R agonists in mice.^40^ The dramatic effects of Lira to reduce liver steatosis make it difficult to observe further improvements given that liver steatosis is comparable to that observed in control chow-fed mice.^28^ Lastly, we utilized a highly sensitive and direct method to assess mRNA signatures related to fibrosis using NanoString technology; however, there may be additional mechanistic insights to uncover utilizing nonbiased, high-throughput -omics technologies such as single-cell RNA sequencing (RNA-seq) and metabolomics.

## STAR★Methods

### Key resources table

**Table:** 

REAGENT or RESOURCE	SOURCE	IDENTIFIER
**Antibodies**		

SMAD 2/3	Cell Signaling Technology	Cat#8685; RRID: AB_10889933
Phospho-SMAD 2/3	Cell Signaling Technology	Cat#8828; RRID: AB_2631089
β-Tubulin	Fisher	Cat#322600; RRID: AB_2533072

**Chemicals, peptides, and recombinant proteins**		

Dextrose	Caledon Laboratory Chemicals	Cat#3260-1-70
Insulin, Human Recombinant	Fisher Scientific	Cat#12585014
Formalin Buffered 10%	ACP Chemicals	Cat#F6000
TRIzol Reagent	ThermoFisher Scientific	Cat#15596026
Sodium Pyruvate (powder)	Sigma-Aldrich	Cat#P2256
DNase I	Roche	Cat#10104159001

**Critical commercial assays**		

RNeasy Mini Kit	Qiagen	Cat#74106
Alanine Transaminase Microplate Assay Kit	Cohesion Biosciences	Cat#CAK1002
Aspartate Transaminase Microplate Assay Kit	Cohesion Biosciences	Cat#CAK1004
Cholesterol E kit	Fujifilm	Cat#999-02601
Non-esterified fatty acid kit (NEFA-HR(2))	Fujifilm	Cat# 999–34691, 991–34891, 993-35191
Triglyceride Kit	Cayman Chemical	Cat#10010303
Ultra-Sensitive Mouse Insulin ELISA kit	Crystal Chem	Cat#90080
Serum Amyloid A kit	R&D Systems	Cat#MSAA00
Secretory Phospholipase A2 kit	Cayman Chemical	Cat#765001-96
CXCL10 kit	ThermoFisher	Cat#
nCounter Fibrosis Panel	NanoString Technologies	Cat#115000388
Pierce BCA Protein Assay Kit	ThermoFisher Scientific	Cat#23225

**Deposited data**		

Human NAFLD livers with different fibrosis stages	Govaere et al.^33^	GEO: GSE135251
Whole liver NanoString data superseries	This study	GEO: GSE240424
Whole liver NanoString data commercial fibrosis kit	This study	GEO: GSE240406
Whole liver NanoString data custom kit	This study	GEO: GSE240409

**Experimental models: Organisms/strains**		

Mouse: C57BL/6J	The Jackson Laboratory	Cat#000664; RRID: AB_IMSR_JAX:000664

**Software and algorithms**		

ImageJ	N/A	https://imagej.nih.gov/ij/
nSolver Analysis Software 4.0	NanoString	https://nanostring.com/products/analysis-solutions/nsolver-advanced-analysis-software/
R version 3.6.0 (R Core Team (2022)	R	https://www.r-project.org/
GraphPad Prism version 9.3.1 (350)	GraphPad	https://www.graphpad.com/scientific-software/prism/

**Other**		

Rodent diet with 40% kcal fat, 20% kcal fructose, 0.02% cholesterol	Research Diets	Cat#D19101102
Accu-Chek Inform II system (glucometer)	Roche	N/A
Accu-Chek Guide (test strips)	Roche	N/A
Specific-pathogen free (SPF) Microisolators	The Jackson Laboratory	N/A
Nikon 90i Eclipse	Nikon	N/A

### Resource availability

#### Lead contact

Further information and request for resources and reagents can be directed to the lead contact, Gregory Steinberg (gsteinberg@mcmaster.ca).

#### Materials availability

This study did not generate any new reagents.

### Experimental model and subject details

#### Diet-induced NASH mouse model

All *in vivo* experiments were approved by the McMaster University Animal Ethics Committee (#21-01-4) and conducted under the Canadian guidelines for animal research. Male mice with a C57BL/6J background were purchased from Jackson Laboratories at 6–7 weeks of age. Mice were housed 3–5 per cage in a controlled environment; 12-h light/dark cycle, given food and water *ad libitum*, and enrichment provided. At 8 weeks of age, mice were moved into specific-pathogen free (SPF) microisolators in a room maintained at ∼29°C and fed a high-fat, high-fructose diet (NASH Diet; ND; 40% fat from mostly palm, 20% fructose, 0.02% cholesterol). Due to fructose being supplemented in the diet, diet was changed every few weeks. 16 weeks later, mice were grouped by matching body weight and adiposity randomly and placed on respective interventional arms. Adiposity was assessed based on time-domain NMR using a Bruker Minispec LF90II. Control mice were continued on diet alone, vehicle-treated mice (Vehicle) were given subcutaneous saline injections every second day 1–2 h before the dark cycle, bempedoic acid-treated mice (BemA) had the drug supplemented in the diet at a dose of 10 mg/kg, liraglutide-treated mice (Lira) were given subcutaneous injections of Victoza diluted in saline to a dose of 70 μg/kg every second day 1–2 h before the dark cycle, and combination-treated mice (Lira+BemA) were given subcutaneous injections of Victoza diluted in saline to a dose of 70 μg/kg every second day 1–2 h before the dark cycle with bempedoic acid supplemented in the diet at a dose of 10 mg/kg. After 9 weeks of treatment, all mice were sacrificed in the fed state between 0900 and 1100 h, using a ketamine/xylazine mixture to sedate mice before collecting blood via cardiac puncture. Mice were presumed dead by exsanguination and cervical dislocation was performed as a secondary measure.

#### Metabolic testing

Metabolic tests were performed between 4 and 9 weeks of intervention, in respective order below. Intraperitoneal glucose (ipGTT; 1.25 g/kg) and insulin (ipITT; 1.3 U/kg) tolerance tests were performed in 6-h fasted mice, with fasting starting at 0700 h and basal values being tested at 1300 h. Intraperitoneal pyruvate tolerance tests (ipPTT; 1.5 g/kg) were performed in 15-h fasted mice, with fasting occurring overnight and basal values being evaluated at 0900hrs. Fasting blood glucose and serums were collected in 6-h fasted mice, mimicking ipGTT and ipITT times. Blood collection for these tests were obtained via tail-knick.

### Method details

#### Liver lipid analysis

Liver fat percentage was assessed based on time-domain NMR using a Bruker Minispec LF90II. Briefly, ∼30–50 mg tissue chips were obtained on dry ice, given 10 min to thaw on ice and given 10 min to equilibrate at room temperature before being placed in biopsy tubes purchased from Bruker. Liver triglycerides were assessed using the Cayman Chemicals Triglyceride Colorometric Assay kit (Item no. 10010303). Briefly, 10–20 mg of frozen liver was immediately homogenized in 400 μL of diluted NP40 substitute assay reagent. The manufacturer’s instructions were followed for all other aspects of the assay.

#### Histology

Tissues were fixed in 10% neutral buffered formalin for 48 h before being stored in 70% ethanol. The medial lobe of the liver was processed, paraffin embedded, serially sectioned, and stained with haemotoxylin and eosin (H&E), Masson’s Trichrome, and picrosirius red (PSR) by the McMaster Immunology Research Center’s core histology facility. Images were acquired by a Nikon 90i Eclipse upright microscope. Liver histology scores were obtained by a blinded pathologist who utilized descriptions as documented by Kleiner and colleagues as their basis (Kleiner et al. 2005). NAFLD activity scores were compiled by the sum of scores: liver steatosis, lobular inflammation, and hepatocellular ballooning, as assessed using H&E-stained slides. Fibrosis scores were obtained by the assessment of both Masson’s Trichrome and PSR-stained slides.

#### RNA isolation and analysis

Liver tissue (∼15 mg) was lysed in 1 mL TRIzol reagent (Invitrogen) using ceramic beads and a Precellys 24 homogenizer (Bertin Technologies). Samples were spun down for 10 min at 12 000 g at 4°C. 200 μL of chloroform was added and shaken vigorously before spinning samples again at same settings. Supernatant was placed in new tubes and an equal amount of 70% ethanol was added then vortexed. Solutions were loaded onto RNeasy columns and manufacturer’s instructions were followed including a DNAse I digestion step (Qiagen).

#### Serum measurements

Serum insulin was assessed in 6-h fasted samples using the manufacturer’s instructions for the Ultra-Sensitive Mouse Insulin ELISA kit (Crystal Chem, Catalog # 90080). Fed serum samples were assessed using the manufacturer’s instructions for: cholesterol E (Fujifilm, No. 999–02601) triglycerides (Cayman Chemicals, Item no. 10010303), non-esterified fatty acids (Fujifilm, NEFA-HR (2), 999–34691, 991–34891, 993–35191), ALT (Cohesion, #CAK1002), AST (Cohesion #CAK1004), Serum Amyloid A (R&D Systems, MSAA00), and sPLA2 (Cayman Chemical, Item No. 765001–96). ProCartaPlex Mouse kits from ThermoFisher were used to measure CRP and CXCL10 on a Bio-Rad Bio-Plex Reader.

#### Immunoblotting

Lysates were prepared in lysis buffer (50 mM HEPES pH 7.4, 150 mM NaCl, 100 mM NaF, 10 Na-pyrophosphate, 5 mM EDTA, 250 mM sucrose, and freshly added 1 mM DTT, and 1 mM Na-orthovanadate, 1% Triton X- and Complete protease inhibitor cocktail (Roche)) and homogenized using ceramic beads and a Precellys 24 homogenizer (Bertin Technologies). Samples were spun down for 10 min at 12 000 g at 4°C and supernatant was taken. Protein concentration was determined via BCA assay, prepared in 4x SDS (sodium dodecyl sulfate) sample buffer at 1 μg/μL, and boiled at 95°C for 5 min. 5 μg of protein was loaded per well and proteins were separated using SDS-polyacrylamide gels electrophoresis (PAGE) (4%–12%) Bis/Tris, in TRIS-glycine-SDS running buffer and electrophoretically transferred to polyvinyl difluoride (PVDF) membranes in 20% methanol TRIS-glycine transfer buffer. Membranes were blocked in 5% BSA in TBST and membranes were probed using antibodies specified in the key resources table.

### Quantification and statistical analysis

#### NanoString gene expression analysis

For NanoString analysis, 4–5 RNA samples per group were inspected by a BioAnalyzer quality control test. The McMaster Genomics Facility ran an nCounter Fibrosis v2 Panel (NanoString Technologies) containing 760 target genes as well as a CustomSet Panel consisting of 22 orthologous mus musculus genes that correspond to the 25-gene NASH severity signature described by Govaere and colleagues.^33^ Gene expression data was normalized and log-transformed prior to differential gene expression analysis and pathway signature score computation using NanoString Technologies’ nSolver 4.0 software (version 4.0.70) and the embedded PLAGE algorithm.

Uniquely regulated genes were defined as differentially expressed genes (FDR <0.05) between control and combination treatment but not monotherapy. Additively regulated genes were defined as differentially expressed genes (FDR <0.05) exhibiting the largest fold change between control and combination treatment compared to monotherapy. Combination specific signature was derived based on the overlap between uniquely and additively regulated genes and differential expression (p value <0.05) between combination treatment and monotherapy. An elastic net regularization model with 10-fold cross validation was used to further identify a subset of genes associated with fibrosis stages >2 in a cohort of 216 human NAFLD/NASH patients using methods implemented in caret v6.0.93 and glmnet v4.1.6.Pathway over-representation was determined by tabulating the pathway annotations associated with genes in the unique and additive gene sets. Statistical significance was computed using Chi-square test.

#### RNA-seq analysis

Patient derived RNA-Seq data was obtained from GEO repository GSE135251 and processed for quality control, alignment, and count as described previously.^33^ Variance stabilizing transformation was applied to compute relative mRNA abundance using DESeq2 v1.36.0.

#### Integrated human and mouse gene expression analysis

Gene expression data derived from NanoString and DESeq2 analyses underwent log-scale and *Z* score transformation prior to integration with human data. PCA and hierarchical clustering using Pearson correlation, as implemented in stats v4.2.2, were applied to integrated gene expression data to assess sample similarity between treatment cohorts and human NASH/NAFLD disease stages.

Multivariate logistic regression, as implemented in the caret v6.0.93, using the combination treatment specific gene signatures and the 25-gene signature reported by Govaere et al. were used to predict advanced fibrosis stages in patients. AUROC scores were computed to assess the predictive performance of each gene signature using methods implemented in pROC v1.18.0.

Classification of patients based on similarity to the combination specific gene signature was computed using Nearest Template Prediction as implemented in GenePattern.^36^ The gene set associated with the similar class is defined as upregulated genes within the combination specific gene signature. The gene set associated with the dissimilar class is defined as downregulated genes within the combination specific gene signature. Only patients with statistically significant classification (p value <0.05) were included in downstream analyses.

ssGSEA was performed as implemented in GenePattern. Log-transformed gene expression along with hallmark gene sets and Aizarani liver cell type gene sets derived from MSIgDB were used as the respective inputs for ssGSEA. Differential ssGSEA scores were computed using T-test followed by FDR adjustment and significance was defined as FDR <0.05.

#### Statistics

All other statistical analyses not previously specified using R packages or GenePattern softwares were performed using GraphPad Prism 9. Values throughout the illustrations are shown as means ± S.E.M. with p values reported in the graphs. Colored bars signify comparisons between groups (with respective colors) and control groups (both ND and ND + Veh). Significance was accepted at p < 0.05 and determined via unpaired t-tests, one-way or repeated-measures two-way ANOVA with Tukey or Sidak’s posthoc, where appropriate. For histological score analysis, a Kruskal-Wallis test or Mann-Whitney tests were used – these are nonparametric tests, with the Kruskal-Wallis test comparing the rank of each column with every other column, and correcting for multiple comparisons using Dunn’s posthoc test. White circles are individual mice per group (n = 8–9 mice/group).

## Data Availability

• Raw and processed Nanostring count data generated in this has been deposited in the NCBI GEO database and is referenced in the key resources table. This paper analyzes data from existing and publicly available RNA sequencing data, which is referenced in the key resources table. • This paper does not report the original code. • Any additional information required to reanalyze the data reported in this work paper is available from the lead contact upon request.

## Supplemental information

Document S1. Figures S1–S4

Table S1. All differentially expressed genes, related to Figure 3

Table S2. Downregulated genes and pathway annotations, related to Figure 3

Table S3. Pathway over-representation analysis of genes uniquely and additively downregulated by combination treatment, related to Figure 3

Document S2. Article plus supplemental information
