# Supplementary material for: Combination of an ACLY inhibitor with a GLP-1R agonist exerts additive benefits on nonalcoholic steatohepatitis and hepatic fibrosis in mice
Source: Cell Rep Med. 2023 Sep 19;4(9):101193. doi: 10.1016/j.xcrm.2023.101193 (PMC10518624; doi:10.1016/j.xcrm.2023.101193)
Supplement: Document S1. Figures S1–S4 [file mmc1.pdf]

Cell Reports Medicine, Volume 4

Supplemental information

Combination of an ACLY inhibitor with a GLP-1R agonist exerts additive benefits on nonalcoholic steatohepatitis and hepatic fibrosis in mice

Eric M. Desjardins, Jianhan Wu, Declan C.T. Lavoie, Elham Ahmadi, Logan K. Townsend, Marisa R. Morrow, Dongdong Wang, Evangelia E. Tsakiridis, Battsetseg Batchuluun, Russta Fayyazi, Jacek M. Kwiecien, Theodoros Tsakiridis, James S.V. Lally, Guillaume Paré, Stephen L. Pinkosky, and Gregory R. Steinberg

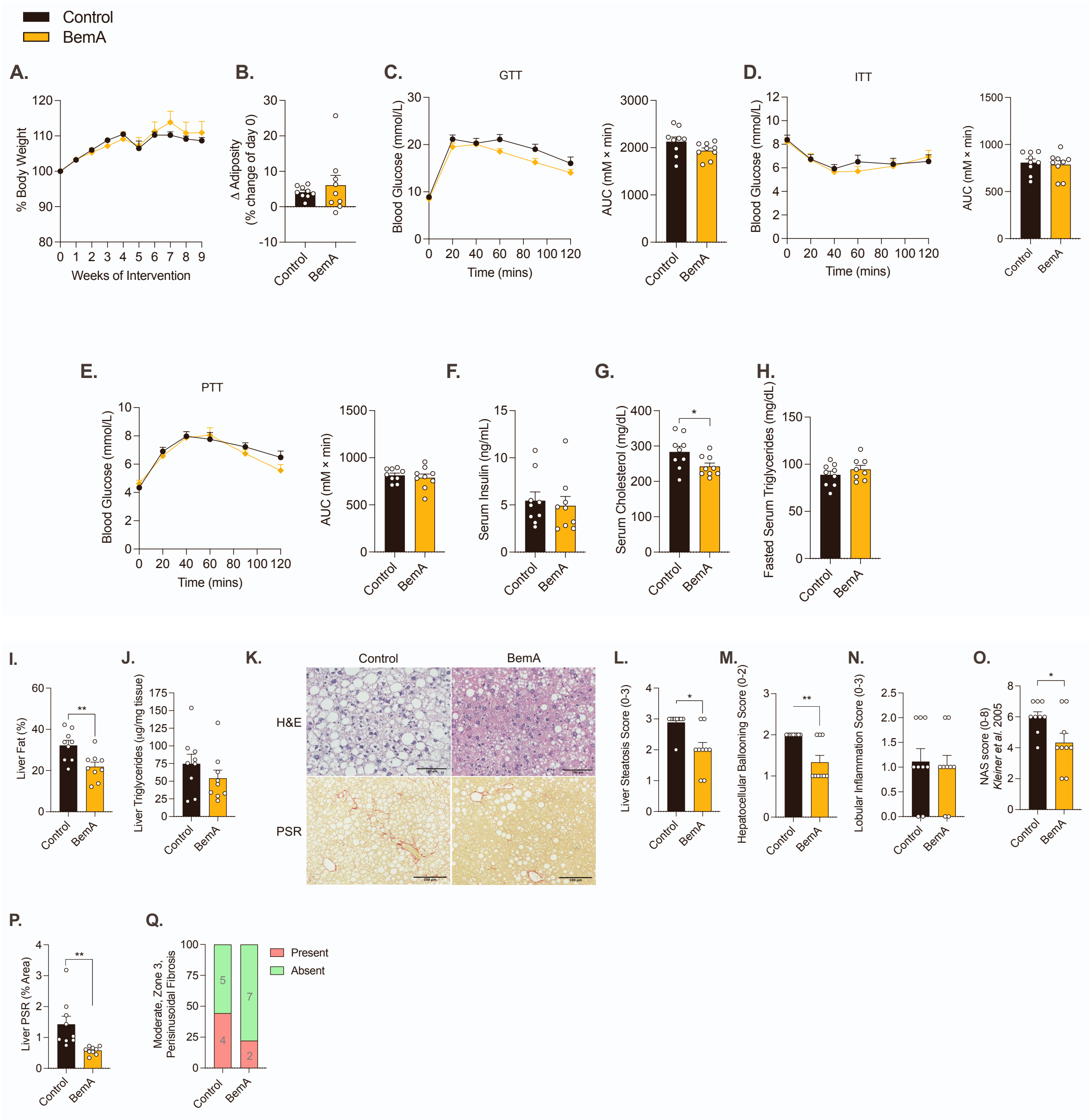

Supplemental Figure 1. Bempedoic acid reduces serum cholesterol, liver steatosis, hepatocellular ballooning, NAFLD activity composite score, and fibrosis independently from body weight, adiposity, and insulin sensitivity in a mouse model of diet-induced NASH. Related to Figures 1 and 2. Percent change in body weight (A) and change in adiposity (post-pre) (B) throughout intervention. Intraperitoneal glucose tolerance test (GTT) (1.25 g/kg) (C) at 4 weeks intervention, ip insulin tolerance test (ITT) (1.3 U/kg) (D) at 4 weeks intervention and ip pyruvate tolerance test (PTT) (1.5 g/kg) (E) at 5 weeks intervention with time plots and area under the curve (AUC). Fasted serum insulin (F) collected via tail-knick near-end of intervention (9 weeks). Fed serum cholesterol (G) from blood collected by cardiac puncture at sacrifice, and fasted serum triglycerides (H). (I) Liver fat percentage as measured by time-domain NMR. (J) Liver triglycerides. (K) Representative micrographs of H&E (top) and picrosirius red (PSR; bottom) stained sections (10x) along with histograms of histological grades of liver steatosis (L), hepatocellular ballooning (M), lobular inflammation (N), and composite NAFLD activity score (NAS) (O), Percent positive PSR area (P) and parts of whole indicating presence of moderate, zone 3 perisinusoidal fibrosis (Q). Data are means ± S.E.M. Black bars signify comparisons between group and control group (ND). Significance was accepted at $p < 0.05$ and determined via unpaired t-test or repeated-measures two-way ANOVA with Sidak posthoc, or, for histological score analysis, a Mann-Whitney test was used, where appropriate. White circles are individual mice per group (n=8-9 mice/group). *P<0.05. ND (or control), ND+BemA (bempedoic acid 10 mg/kg in diet).

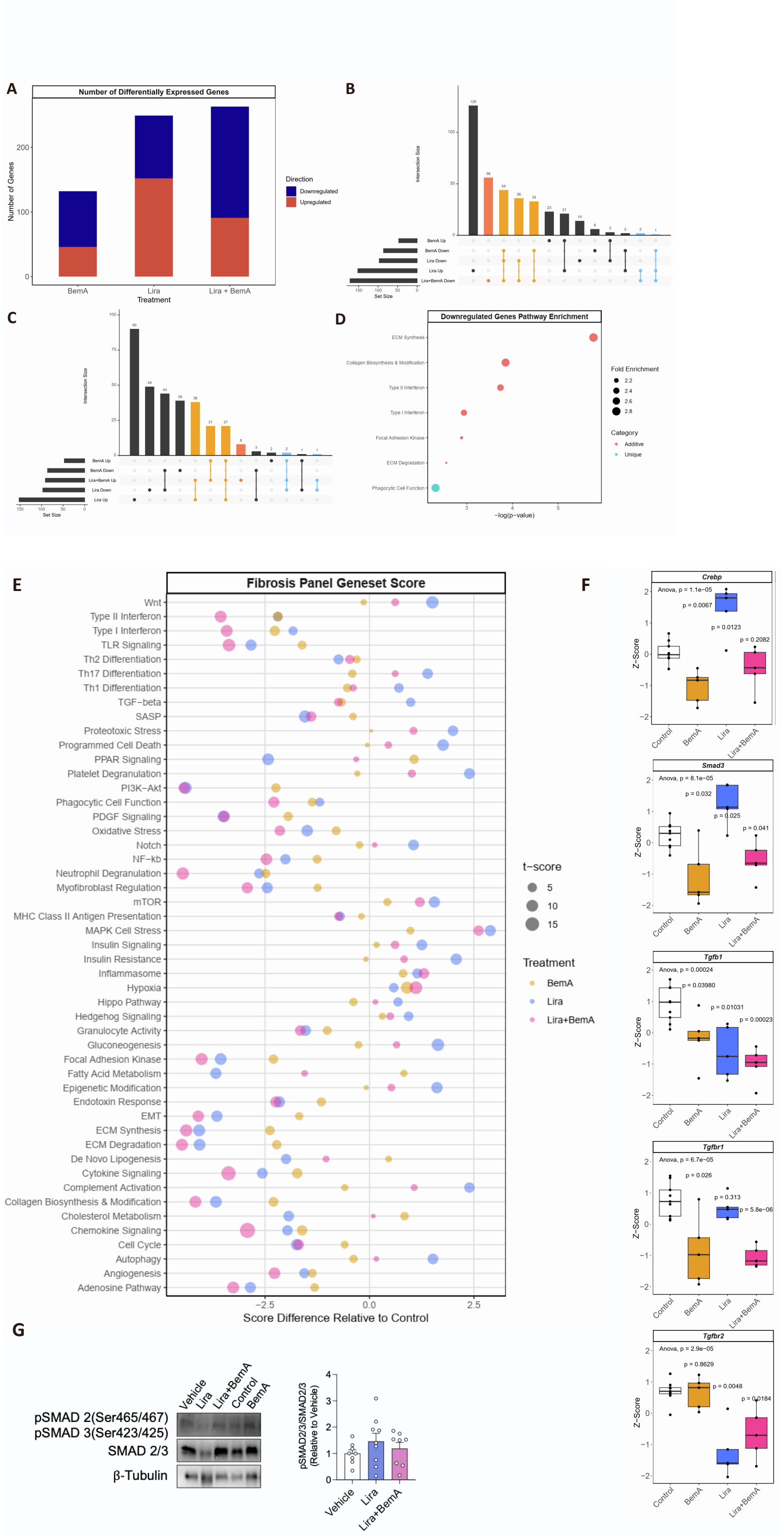

Supplemental Figure 2. Targeted gene expression, gene set analysis, and TGFβ-related gene expression analysis. Related to Figure 3. (A) Number of differentially expressed genes in each treatment group. (B) Overlap between significantly downregulated and (C) upregulated genes by combination treatment and genes differentially expressed in all other treatment groups. Red indicates genes uniquely regulated by combination treatment. Orange indicates genes up or downregulated in all treatment groups. Blue indicates genes upregulated by monotherapy and downregulated by combination treatment. (D) Over-represented gene set annotations associated with additively and uniquely downregulated genes by combination treatment. (E) Signature scores of all gene sets in the Nanostring nCounter Fibrosis v2 Panel. (F) Expression of genes involved in TGFβ pathway.(G) Immunoblotting of phosphorylated SMAD 2 (Ser465/467)/SMAD 3 (Ser423/425) over total SMAD 2/3. Black/white circles are individual mice per group (n=5-9 for gene set analysis, 8-9 for immunoblotting). Boxplots show median and interquartile range, trailing lines represent 95% confidence interval. Difference between groups were assessed by one-way ANOVA followed by Dunnett's post hoc test using the control group as the reference level. Significance was accepted at $P < 0.0033$ to correct for Bonferroni multiple hypothesis testing.

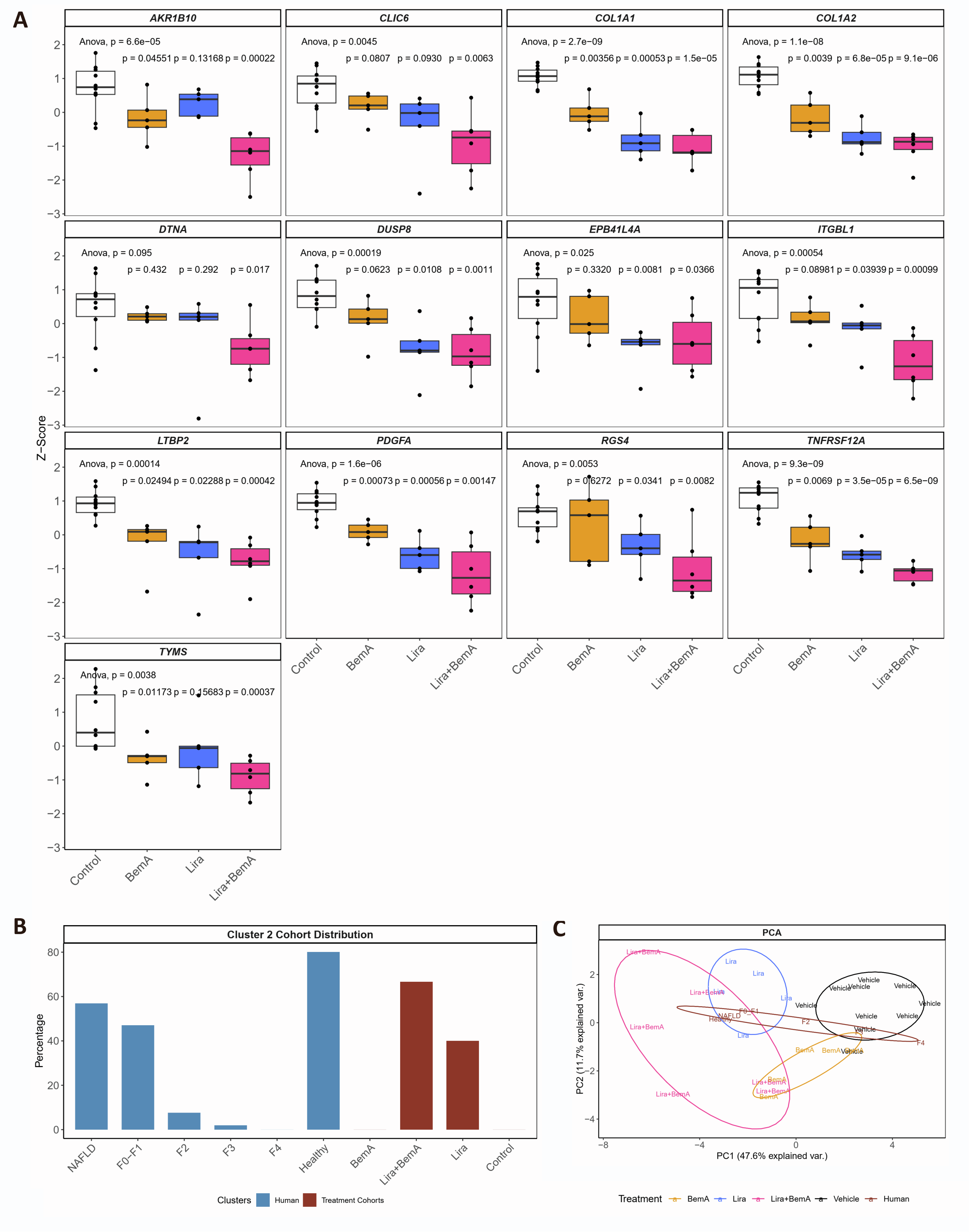

Supplemental Figure 3. Combination treatment reverses the expression of prognostically significant genes involved in NASH progression. Related to Figure 4. (A) Scaled expression of differentially expressed and prognostically significant orthologous genes involved in NASH progression in healthy, NASH/NAFLD patients and experimental cohorts. (B) Distribution of disease stages and treatment types in cluster II. (C) PCA of human NASH/NAFLD patients and experimental cohorts based on scaled gene expression. Black circles represent individual mice per group (n = 5-9 mice/group). Boxplots show median and interquartile range, trailing lines represent 95% confidence interval. Difference between groups were assessed by one-way ANOVA followed by Dunnett's post hoc test using the control group as the reference level. Nominal significance was accepted at $P < 0.05$ and at $P < 0.002$ when corrected for Bonferroni multiple hypothesis testing.

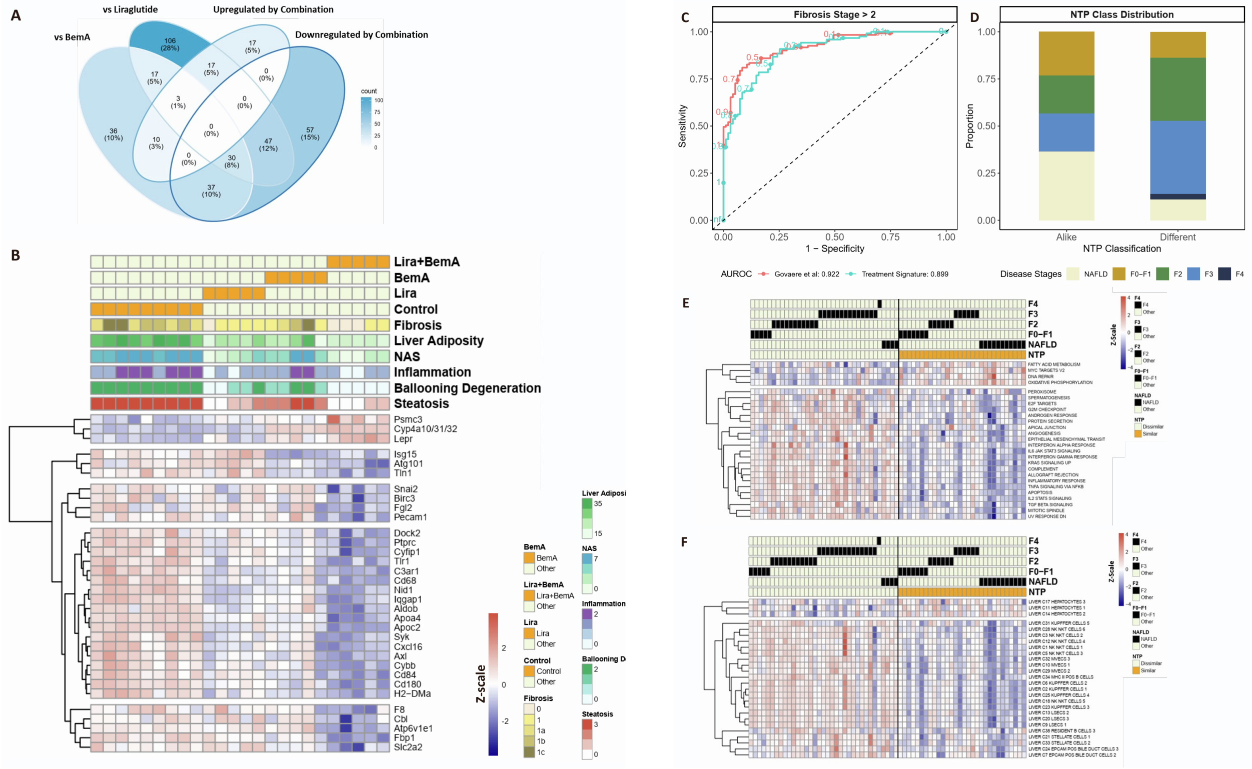

Supplemental Figure 4. Development of a combination treatment specific gene signature for supervised classification, and clinical outcome and gene expression profile associated with combination treatment specific gene signature. Related to Figure 4. (A) Comparison between genes differentially regulated by combination treatment compared to both control and monotherapies. (B) Expression of the 33-gene signature in treatment cohorts. n=5-9/group. (C) ROC of multivariate logistic regression models using the combination treatment specific gene signature, or the 25-gene signature previously reported by Govaere et al. for the prediction of advanced fibrosis among human NASH/NAFLD patients. (D) Distribution of disease stages among human derived samples classified as either alike or different based on NTP, using the combination treatment specific signature as the reference. (E) Sample level ssGSEA scores of hallmark gene sets or (F) liver cell type gene sets between samples classified as either alike or different based on NTP. n=5-9/group.
